# Inhibition of all-*TRANS*-retinoic acid metabolism by R116010 induces antitumour activity

**DOI:** 10.1038/sj.bjc.6600056

**Published:** 2002-02-12

**Authors:** J Van heusden, R Van Ginckel, H Bruwiere, P Moelans, B Janssen, W Floren, B J van der Leede, J van Dun, G Sanz, M Venet, L Dillen, C Van Hove, G Willemsens, M Janicot, W Wouters

**Affiliations:** Department of Oncology Discovery Research, Johnson & Johnson Pharmaceutical Research & Development, Turnhoutseweg 30, B-2340 Beerse, Belgium; Department of Metabolic Disorders, Johnson & Johnson Pharmaceutical Research & Development, Turnhoutseweg 30, B-2340 Beerse, Belgium; Department of Medicinal Chemistry, Johnson & Johnson Pharmaceutical Research & Development, Val-de-Reuil, France; Drug Evaluation, Johnson & Johnson Pharmaceutical Research & Development, Turnhoutseweg 30, B-2340 Beerse, Belgium

**Keywords:** RA, metabolism, inhibitor, CYP26A1, R116010

## Abstract

All-*trans*-retinoic acid is a potent inhibitor of cell proliferation and inducer of differentiation. However, the clinical use of all-*trans*-retinoic acid in the treatment of cancer is significantly hampered by its toxicity and the prompt emergence of resistance, believed to be caused by increased all-*trans*-retinoic acid metabolism. Inhibitors of all-*trans*-retinoic acid metabolism may therefore prove valuable in the treatment of cancer. In this study, we characterize R116010 as a new anticancer drug that is a potent inhibitor of all-*trans*-retinoic acid metabolism. *In vitro*, R116010 potently inhibits all-*trans*-retinoic acid metabolism in intact T47D cells with an IC_50_-value of 8.7 nM. In addition, R116010 is a selective inhibitor as indicated by its inhibition profile for several other cytochrome P450-mediated reactions. In T47D cell proliferation assays, R116010 by itself has no effect on cell proliferation. However, in combination with all-*trans*-retinoic acid, R116010 enhances the all-*trans*-retinoic acid-mediated antiproliferative activity in a concentration-dependent manner. *In vivo*, the growth of murine oestrogen-independent TA3-Ha mammary tumours is significantly inhibited by R116010 at doses as low as 0.16 mg kg^−1^. In conclusion, R116010 is a highly potent and selective inhibitor of all-*trans*-retinoic acid metabolism, which is able to enhance the biological activity of all-*trans*-retinoic acid, thereby exhibiting antitumour activity. R116010 represents a novel and promising anticancer drug with an unique mechanism of action.

*British Journal of Cancer* (2002) **86**, 605–611. DOI: 10.1038/sj/bjc/6600056 www.bjcancer.com

© 2002 Cancer Research UK

## 

All-*trans*-retinoic acid (RA) is a naturally occurring retinoid that is well-known to inhibit cell proliferation and to induce differentiation (Hong and Itri, 1994; Moon *et al*, 1994). However, the clinical use of RA in the treatment of cancer is significantly hampered by the prompt emergence of resistance, which is believed to be caused by increased RA metabolism (Muindi *et al*, 1992, 1994; Lee *et al*, 1993; Miller, 1998). RA is metabolized by CYP26A1, an inducible cytochrome P450-dependent enzyme, that inactivates RA by 4-hydroxylation of the β-ionone ring (White *et al*, 1997). This tightly-controlled negative feedback mechanism limits the availability of RA and thereby also its biological activity. Therefore, increasing levels of RA by inhibition of its metabolism might prove an innovative approach to cancer treatment.

Previously, we have identified liarozole-fumarate as an inhibitor of RA metabolism (Wouters *et al*, 1992). *In vitro*, liarozole-fumarate has been shown to enhance both the antiproliferative and differentiation-inducing activity of RA (Wouters *et al*, 1992; Van heusden *et al*, 1996, 1998). *In vivo,* liarozole-fumarate increases endogenous RA levels both in plasma and tissues (Smets *et al*, 1995). In several preclinical tumour models, liarozole-fumarate has been shown to exhibit antitumour activity (Van Ginckel *et al*, 1990; Dijkman *et al*, 1994; Smets *et al*, 1995), linked to increased levels of RA within the tumour (Smets *et al*, 1995). In cancer patients, liarozole-fumarate has been shown to increase the half-life of orally administered RA and 13-*cis*-retinoic acid (Westarp *et al*, 1993; Achkar *et al*, 1994; Miller *et al*, 1994). However, one of the limitations to the use of liarozole-fumarate was its lack of specificity. It was shown to inhibit also other cytochrome P450-mediated reactions (Vanden Bossche, 1992) and affect plasma hormone levels in volunteers (Bruynseels *et al*, 1990).

This lack of specificity might partly explain the limited risk benefit ratio observed in cancer patients. A more potent and selective inhibitor is therefore required to improve the clinical efficacy of inhibitors of RA metabolism. In the present study, we identify R116010 ( Figure 1

**Figure 1:**
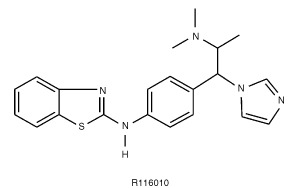
Chemical structure of R116010, [S-(R*,R*)]-N-[4-[2-(dimethylamino)-1-(1H-imidazole-1-yl)propyl]-phenyl]-2-benzothiazolamine.

) as a highly potent and selective second-generation inhibitor of RA metabolism that induces antitumour activity.

## MATERIALS AND METHODS

### Drug and chemicals

R116010, [S-(R*,R*)]-N-[4-[2-(dimethylamino)-1-(1*H*-imidazole-1-yl)propyl]-phenyl]-2-benzothiazolamine, and liarozole-fumarate, ±-5-[(3-chlorophenyl)(1*H*-imidazole-1-yl)methyl]-1*H*-benzimidazole (E)-2-butenedioate (2:3) were synthesized at the Department of Medicinal Chemistry (Johnson & Johnson Pharmaceutical Research & Development, Val-de-Reuil, France). Compounds were dissolved to an initial concentration of 10 mM in DMSO. Further dilutions were made in culture medium. [11, 12-^3^H(N)]RA (30 Ci mmol^−1^) was obtained from NEN Life Science Products (Boston, MA, USA). Unlabelled RA was purchased from Serva (Heidelberg, Germany) and dissolved to an initial concentration of 4 mM in ethanol. Further dilutions were made in culture medium. All-*trans*-retinoic acid was used in a dark-room with yellow illumination. Final solvent concentrations in the *in vitro* assays were always less than 0.5% (v/v). This solvent concentration had no effect in the respective assays.

### Cell culture

Human T47D breast cancer cells – purchased from the American Type Culture Collection (Rockville, MD, USA) – were cultured in RPMI 1640 medium supplemented with 10% (v/v) FBS, 2 mM L-glutamine, 50 μg ml^−1^ gentamicin, and 10 μg ml^−1^ insulin (all reagents from Life Technologies, Gent, Belgium). Cells were grown in a humidified incubator (5% CO_2_, 95% air) at 37°C and were *Mycoplasma*-free as tested by the *Mycoplasma* T.C. kit (Gen-Probe Incorporated, CA, USA).

### Microcolumn assay for RA metabolism

RA metabolism was quantitatively determined using the microcolumn assay as described previously (Krekels *et al*, 1997). Briefly, T47D cells were pretreated for 16 h with 1 μM RA to induce RA metabolism. Cells were then washed twice with culture medium, harvested and resuspended at 4×10^6^ cells ml^−1^. An aliquot of this cell suspension (450 μl) was incubated for 90 min in the presence of 0.1 μM [11, 12-^3^H(N)]RA, after which 2 ml acetonitrile was added. After centrifugation for 10 min at 780 **g**, the resulting deproteinized supernatant was acidified with 2.5 ml of 40 mM acetic acid and applied to a 3 ml C18 Bond Elut LRC column (Varian, Harbor City, CA, USA; pretreated with 4 ml distilled water) under a vacuum of 127 mmHg using VAC ELUT SPS-24 and the effluent was collected. The column was eluted with 1 ml 40% acetonitrile and the effluent was collected in the same vial. Radioactivity in the collected effluent, containing the polar metabolites, was determined in a Packard Tri-carb 4530 liquid scintillation analyzer. Optiphase ‘Hi Safe II’ (Wallac, Milton Keynes, UK) was used as a scintillator. Data are presented as mean±s.d. of three or four independent experiments. The IC_50_-values were calculated by non-linear regression analysis using SigmaPlot 4.01 software.

### HPLC analysis

The T47D cell suspension was prepared as described above and 450 μl was incubated with 0.1 μM [11, 12-^3^H(N)]RA for 90 min. After centrifugation for 10 min at 780 **g**, the supernatant was analyzed for the presence of RA metabolites. Reverse-phase HPLC analysis was carried out as described previously (Van heusden *et al*, 1998).

### *In vitro* cytochrome P450 isozyme specificity

Metabolism of RA by CYP26A1 was measured in microsomes of T47D human breast cancer cells that were pretreated for 16 h with 1 μM RA to induce RA metabolism. Microsomes were prepared exactly as described by Han and Choi (1996), and RA metabolism was measured as described by Van Wauwe *et al* (1994). The conversion of androstenedione to estrone by aromatase (CYP19) in human placental microsomes, the conversion of 17-hydroxy-20-dihydroprogesterone to testosterone by 17,20-lyase (CYP17) in rat testicular S10 fractions, and the 2α-, 7α-, 6β-, and 16β-hydroxylation of testosterone (CYP2C11, CYP2A1, CYP3A, CYP2B1/2) in rat liver microsomes were carried out as described (Vanden Bossche *et al*, 1990).

### Proliferation experiments (3-(4,5-dimethylthiazol-2-yl)-2,5-diphenyl-2*H*-tetrazolium bromide assay)

T47D cells were seeded in Falcon® 96-well cell culture plates (Life Technologies, Merelbeke, Belgium) at 2500 cells per well in a total volume of 150 μl. Cells were allowed to adhere to plastic for 24 h. Drugs and/or medium were added to a final volume of 200 μl (day 0). Cells were grown under these conditions for 7 days, with renewal of medium and drugs on days 2 and 5. On day 7, medium was renewed and 25 μl MTT-solution was added, and cells were further incubated for 2 h in a cell incubator. The medium was aspirated and 25 μl Sorensen glycine buffer (0.1 M glycine, 0.1 M NaCl; pH 10.5) was added together with 100 μl DMSO to solubilize the blue MTT-formazan product. After shaking for 10 min on a microplate shaker, the absorbance at 540 nm was determined using a E_max_ 96-well spectrophotometer (DPC, Grimbergen, Belgium). Data are presented as mean±s.d. of three or four independent experiments. Within an experiment, the result of each experimental condition is the mean of six replicate wells. The IC_50_-values were calculated by non-linear regression analysis using SigmaPlot 4.01 software.

### RT*–*PCR

T47D cells were seeded in Nunc™ 6-well plates at a concentration of 2×10^6^ cells per well in 5 ml medium. Cells were allowed to adhere to plastic for 24 h. After indicated periods of time, total RNA was isolated using UltraSpec-II (Biotecx Laboratories, TX, USA) according to manufacturer's instructions. Three micrograms of total RNA was heated at 65°C for 5 min and rapidly cooled on ice. Then, 1500 ng oligo d(T)_12–18_ primer (Life Technologies, MD, USA) was added and cDNA was synthesized using 200 U μl^−1^ M-MLV RT (Life Technologies, MD, USA) in a RT reaction buffer (Life Technologies, MD, USA) supplemented with 20 U μl^−1^ RNAsin (Life Technologies, MD, USA), and 500 μM each dNTP (Perkin Elmer, CA, USA). The 60 μl reaction mixture was incubated at 37°C for 1 h, followed by a 4 min incubation at 90°C to inactivate the RT enzyme. Two microlitres of the RT reaction mix was used for the PCR reaction. Amplification was performed in a total volume of 50 μl containing 2 μl cDNA, 5 μl 10×PCR reaction buffer (Boehringer Mannheim, Germany), 1 μl sense primer (20 μM), 1 μl antisense primer (20 μM), 4 μl dNTPs (each at a concentration of 2.5 mM; Perkin Elmer, CA, USA) and 0.25 μl AmpliTaq Gold (5 U μl^−1^; Perkin Elmer, CA, USA). AmpliTaq Gold was activated by a 10 min incubation at 94°C. The samples were then subjected to 30 or 24 cycles of amplification for CYP26A1 or β_2_-microglobulin, respectively. Each cycle consisted of 1 min of denaturation at 92°C, 1 min of annealing at 55°C (CYP26A1) or 50°C (β_2_-microglobulin), and 2 min of extension at 72°C. At the end of the incubation an extra extension step was included (10 min at 72°C). Ten microlitres of the PCR products were separated on a 1.3% (w/v) agarose gel, and visualized by UV light illumination after ethidium bromide staining. Specific primers were obtained from Eurogentec (Seraing, Belgium). Their sequences were as follows: CYP26A1 sense primer 5′-GCTGAAGAGTAAGGGTTTAC-3′, and antisense primer 5′-CTTGGGAATCTGGTATCCAT-3′ (yielding a PCR-product of 184 bp); β_2_-microglobulin sense primer 5′-ACCCCCACTGAAAAAGATGA-3′, and antisense primer 5′-ATCTTCAAACCTCCATGATG-3′ (yielding a PCR product of 114 bp).

### TA3-Ha mammary carcinoma model

Oestrogen-independent TA3-Ha murine mammary carcinoma cells were grown by weekly intraperitoneal passage *in vivo*. Cells (1.5×10^6^) were injected intraperitoneally in syngeneic A/J mice. After 1 week, the peritoneum was rinsed with sterile saline and the cells further diluted in MEM-Rega3 cell culture medium (Life Technologies, Merelbeke, Belgium). The next *in vivo* passages (1×10^4^ cells) were done in allogeneic C3D2F1 mice, but every 5th passage syngeneic A/J mice were used as the host. For the current experiments, cells were used between *in vivo* passage 10–20. Mice were subcutaneously inoculated with 1.5×10^6^ murine mammary carcinoma TA3-Ha cells at day 0 and randomized into the different experimental groups. Each group consisted of 10 animals for experiments with R116010 and of six animals for experiments with RA. All treatments were performed by oral gavage. Different doses of R116010 (1.25, 0.63, 0.31, 0.16, 0.08 mg kg^−1^ per dosage) and RA (5, 2.5, 1.25 mg kg^−1^ per dosage) were administered on a twice-daily treatment regimen given from day 1 until day 21. The control groups were given the vehicle 20% 4-OH-β-cyclodextrine in sterile saline. Mice were daily examined for adverse drug effects. At the end of the experiments, subcutaneous tumours were excised 24 h after the last treatment and weighed to validate the antitumoural effects of the treatment.

Data are represented as box plots showing median group value, 25/75th percentile, 10/90th percentile and outliers. Groups were statistically compared to the vehicle-treated groups using the Mann–Whitney *U*-test. Significance was defined at the level of *P*<*0.05*.

All animal experiments have been carried out with ethical committee approval. The ethical guidelines that were followed meet the standards required by the UKCCCR Guidelines.

## RESULTS

### R116010 is a potent inhibitor of RA metabolism

**Figure 2:**
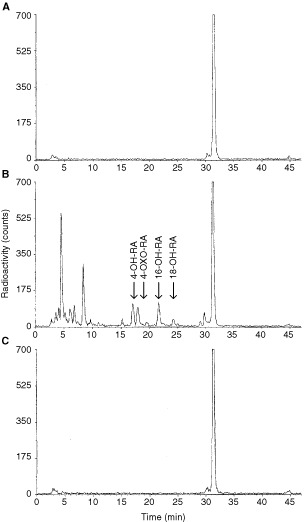
Reverse-phase HPLC analysis of radiolabelled RA metabolites formed in T47D human breast cancer cells. T47D human breast cancer cells were cultured under basal culture conditions (**A**) or pretreated with 1 μM RA (**B**, **C**). Then, cells were collected and incubated with 0.1 μM [^3^H]RA, either in the absence (**A**, **B**) or presence (**C**) of 1 μM R116010. Supernatants were analyzed by reverse-phase HPLC (10).

Human T47D breast cancer cells, cultured under control conditions, are unable to metabolize RA into more polar metabolites ( Figure 2A

). After pretreatment for 16 h with 1 μM RA, T47D cells show extensive RA metabolism (Figure 2B), converting RA into highly polar metabolites (retention time: 3–10 min) and several metabolites with intermediate polarity (retention time: 15–25 min), including 4-OH-RA, and 16-OH-RA. RA metabolism is completely inhibited by R116010 at a concentration of 1 μM (Figure 2C).

**Figure 3:**
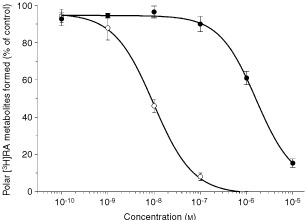
Inhibition of RA metabolism by R116010 and liarozole-fumarate. T47D human breast cancer cells were pretreated with 1 μM RA to induce RA metabolism. Concentration-response curves showing the inhibition by R116010 (○) and liarozole-fumarate (•) were determined using the microcolumn assay as described in Materials and Methods. Results are presented as mean±s.d. for R116010 (*n*=4) and liarozole-fumarate (*n*=3).

A concentration-response curve ( Figure 3

), as measured with the quantitative microcolumn method, shows that R116010 inhibits the formation of polar [^3^H]RA metabolites with a calculated IC_50_-value of 8.7±1.4 nM. In comparison, R116010 is over 100-fold more potent than liarozole-fumarate, a previously identified inhibitor of RA metabolism, that inhibits RA metabolism with an IC_50_-value of 1.4 μM (Figure 3).

### Auto-induction of RA metabolism correlates with induction of CYP26A1

**Figure 4:**
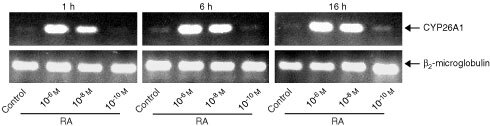
RT–PCR analysis of CYP26A1 mRNA expression after treatment with RA. T47D human breast cancer cells were treated with various concentrations of RA for indicated time periods. Thereafter, total RNA was prepared and subjected to RT–PCR. Amplified products were analyzed by agarose gel electrophoresis with ethidium bromide staining.

CYP26A1 is a cytochrome P450-dependent enzyme that specifically metabolizes RA (White *et al*, 1997). Human T47D breast cancer cells, cultured under control conditions, barely express detectable CYP26A1 mRNA levels ( Figure 4

). CYP26A1 mRNA expression levels can be induced in a concentration- and time-dependent manner by treatment with RA (Figure 4). The expression levels of CYP26A1 closely correlate with the observed enzyme activity (Figure 2B and data not shown).

### R116010 is a selective inhibitor of RA metabolism

The selectivity of R116010 towards CYP26A1 was tested by determining its inhibition profile against other cytochrome P450-mediated reactions. As shown in Table 1

**Table 1:**
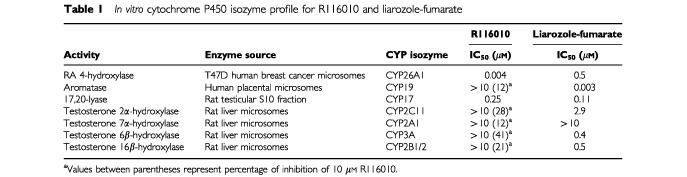
*In vitro* cytochrome P450 isozyme profile for R116010 and liarozole-fumarate

, R116010 inhibits CYP26A1-mediated RA metabolism in microsomes from T47D cells with an IC_50_-value of 4 nM. In contrast, even at high micromolar concentrations (10 μM), R116010 barely inhibits other cytochrome P450-mediated reactions, except for limited activity against 17,20-lyase (IC_50_=0.25 μM).

In comparison, liarozole-fumarate inhibits CYP26A1-mediated RA metabolism at 100-fold higher concentrations (IC_50_=0.5 μM) (Table 1). At this concentration liarozole-fumarate also clearly inhibits other cytochrome P450-mediated reactions (Table 1).

### R116010 enhances the antiproliferative activity of RA

**Figure 5:**
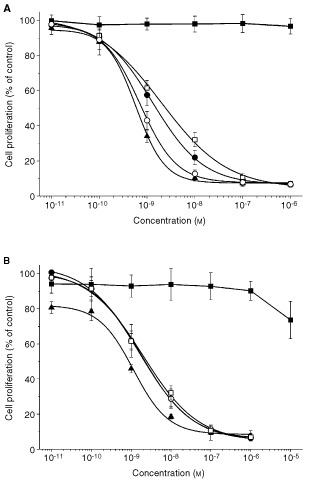
Concentration-response curves showing the antiproliferative effects of (**A**) RA (□), R116010 (▪) and RA in combination with R116010 (•: 0.01 μM; ○: 0.1 μM; ▴: 1 μM) or (**B**) RA (□), liarozole-fumarate (▪) and RA in combination with liarozole-fumarate (•: 0.1 μM; ○: 1 μM; ▴: 10 μM) in T47D cells. Cell proliferation was measured after 7 days using a MTT-based assay as described in detail in Materials and Methods. Results are presented as mean±s.d. for R116010 (*n*=4) and liarozole-fumarate (*n*=3).

RA inhibits T47D cell proliferation in a concentration-dependent manner ( Figure 5A,B

) with a calculated IC_50_-value of 2.0±0.5 nM (Table 2). R116010 by itself has no effect on T47D cell proliferation (Figure 5A). However, in combination with RA, R116010 enhances the antiproliferative activity of RA in a concentration-dependent manner (Figure 5A). At a concentration of 0.01 μM R116010 enhances the antiproliferative activity of RA by 1.25-fold (IC_50_-value=1.6±0.6 nM), at 0.1 μM R116010 by 2.6-fold (IC_50_-value=0.77±0.18 nM) and at 1 μM R116010 by three-fold (IC_50_-value=0.62±0.19 nM).

In contrast, liarozole-fumarate, tested up to a concentration of 10 μM, is unable to enhance the antiproliferative activity of RA (Figure 5B). At this concentration (10 μM), liarozole-fumarate by itself decreases T47D cell proliferation by approximately 25% (Figure 5B). This decrease in T47D cell proliferation at 10 μM explains the apparent enhancement of RA activity, which is actually the effect of liarozole-fumarate alone (Figure 5B).

### R116010 inhibits the growth of murine TA3-Ha mammary tumours* in vivo*

**Figure 6:**
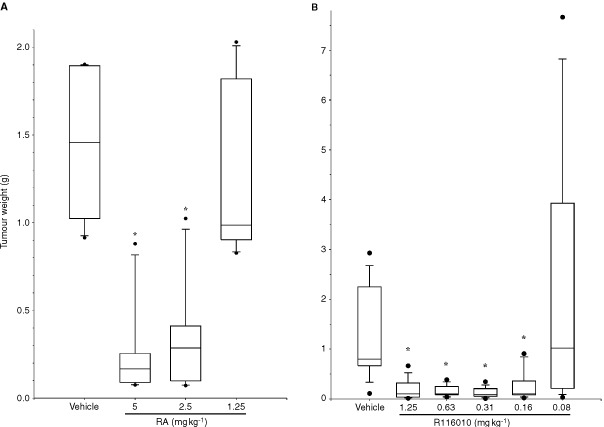
*In vivo* antitumour activity of RA (**A**) and R116010 (**B**) in the murine TA3-Ha tumour model. TA3-HA mammary carcinoma cells (1.5×10^6^) were subcutaneously inoculated in mice. RA (**A**) or R116010 (**B**) was given by oral treatment twice daily from day 1 until day 21. Tumours were excised 24 h after the last treatment and weighed. Data are represented as box plots showing median group value, 25/75th percentile, 10/90th percentile and outliers. Groups were statistically compared to the vehicle-treated groups using the Mann–Whitney *U*-test. Statistical significance was defined at the level of *P<*0.05*.

Mice subcutaneously inoculated with oestrogen-independent TA3-Ha cells were treated twice-daily with RA ( Figure 6A

) or R116010 (Figure 6B) from day 1 until day 21. As shown in Figure 6A, RA inhibits TA3-Ha tumour growth in a dose-dependent manner. The lowest active dose is 2.5 mg kg^−1^. At a dose of 5 mg kg^−1^, mice lost 5% of body weight (data not shown) and toxicity was observed, which consisted mainly of hair loss, and impaired movement due to bone fractures.

R116010 potently inhibits TA3-Ha tumour growth at doses as low as 0.16 mg kg^−1^ (Figure 6B). R116010 is approximately 60-fold more potent than liarozole-fumarate which in this model inhibits tumour growth only at 10 mg kg^−1^ (data not shown). Toxicity, related to hypervitaminosis A, was observed at a dose of 5 mg kg^−1^ for R116010 and at 20 mg kg^−1^ for liarozole fumarate. At these doses hair loss and bone fractures were observed, and body weight decreased by ∼15% (data not shown).

## DISCUSSION

The prompt emergence of resistance to RA treatment is a major hurdle in the clinical development of RA (Muindi *et al*, 1992; Miller, 1998). Drugs which are able to inhibit RA metabolism may therefore prove valuable in the treatment of cancer. In this study, we have identified R116010 as a highly potent and selective inhibitor of RA metabolism.

Human breast T47D carcinoma cells are known to have an inducible RA metabolism (Han and Choi, 1996; Van der Leede *et al*, 1997). The capacity to metabolize RA closely correlates with the expression level of CYP26A1, a cytochrome P450-dependent enzyme that specifically metabolizes RA (White *et al*, 1997). In untreated T47D cells, which are unable to metabolize RA, CYP26A1 expression is barely detectable. Treatment with RA rapidly induces the capacity to metabolize RA (Han and Choi, 1996), which is correlated with a strong induction of CYP26A1, in agreement with a previous report (Sonneveld *et al*, 1998). In intact T47D cells, R116010 potently inhibits RA metabolism with an IC_50_-value of 8.7 nM. As such, R116010 is more than 100-fold more potent than liarozole-fumarate, a previously identified inhibitor of RA metabolism (Wouters *et al*, 1992).

As well as being a more potent inhibitor of RA metabolism, R116010 is also more selective as compared to liarozole-fumarate. Although our *in vitro* data suggest that R116010 might affect androgen levels by inhibiting 17,20-lyase, no change on blood hormone levels could be detected in rats treated with a high dose (10 mg kg^−1^) of R116010 (data not shown). Liarozole-fumarate is much less specific. It is a potent aromatase inhibitor (Bruynseels *et al*, 1990), and in addition it also inhibits other cytochrome P450-mediated reactions, thereby affecting hormone levels both in animals and in humans (Bruynseels *et al*, 1990). Taken together, these data suggest that R116010 is less likely to produce adverse side effects.

In T47D cell proliferation experiments, R116010 enhances the antiproliferative activity of RA in a concentration-dependent manner. Concentrations effective in enhancing the biological activity of RA, are identical to the concentrations that are needed to inhibit RA metabolism in intact cells. Therefore, these data support the hypothesis that R116010 enhances the biological activity of RA through inhibition of RA metabolism. Liarozole-fumarate is unable to enhance the antiproliferative activity of RA in T47D cells. This is in contrast to our previous data, which clearly showed that liarozole-fumarate is able to enhance both the antiproliferative and differentiation-inducing activity of RA in MCF-7 human breast cancer cells (Wouters *et al*, 1992; Van heusden *et al*, 1996). This apparent contradiction might be explained by the fact that T47D cells are known to metabolize RA at a much higher rate than MCF-7 cells (Van der Leede *et al*, 1997; unpublished results) and that liarozole-fumarate is therefore not potent enough to effectively inhibit RA metabolism in T47D cells.

*In vivo,* R116010 has been shown to inhibit the growth of orthotopically implanted androgen-independent rat prostatic Dunning R3327/PIF-1 carcinoma cells (Van Ginckel *et al*, 1999), and of subcutaneous mouse Lewis lung (3LL) tumours (Smets *et al*, manuscript in preparation). In this study, the *in vivo* activity of R116010 has been evaluated in the murine oestrogen-independent TA3-Ha mammary carcinoma model. An oestrogen-independent model was chosen to exclude any hormonal mechanism of action. In addition, TA3-Ha cells are known to actively metabolize RA (Garrabrant and End, 1995) and TA3-Ha tumours are sensitive to RA treatment (Wouters *et al*, 1990). Indeed, treatment with RA inhibits TA3-Ha tumour growth in a dose-dependent manner. However, keeping in mind that the lowest active dose is 2.5 mg kg^−1^ and that the maximum tolerable dose is 5 mg kg^−1^, RA does not show a real therapeutic window. Similarly, liarozole-fumarate has no therapeutic window since a dose of 10 mg kg^−1^ has to be used to induce antitumour activity, and the maximum tolerated dose is 20 mg kg^−1^ (data not shown). In sharp contrast, R116010 inhibits TA3-Ha tumour growth at doses as low as 0.16 mg kg^−1^. With a maximum tolerated dose of 5 mg kg^−1^, R116010 exhibits a 30-fold therapeutic window. We speculate that this larger therapeutic window is due to the intratumoural action of R116010, thereby increasing intracellular levels of RA, while RA mainly remains in the circulation, and much higher doses are needed to produce a biological response. Given that R116010 has a broader therapeutic window, one can argue that R116010 may be a more effective anticancer drug. The observation of retinoid-like side effects at higher doses reinforces the hypothesis that R116010 exerts its antitumour activity via RA.

In conclusion, R116010 has been identified as a potent and selective inhibitor of RA metabolism. Inhibition of RA metabolism leads to an enhancement of the antiproliferative activity of RA both *in vitro* and *in vivo*. Inhibitors of RA metabolism may therefore represent a novel class of anticancer agents.
